# Using SNP Weights Derived From Gene Expression Modules to Improve GWAS Power for Feed Efficiency in Pigs

**DOI:** 10.3389/fgene.2019.01339

**Published:** 2020-01-21

**Authors:** Brittney N. Keel, Warren M. Snelling, Amanda K. Lindholm-Perry, William T. Oliver, Larry A. Kuehn, Gary A. Rohrer

**Affiliations:** USDA, ARS, U.S. Meat Animal Research Center, Clay Center, NE, United States

**Keywords:** constrained tensor decomposition, gene expression, clustering, feed efficiency, swine, GWAS, weighted SNP

## Abstract

The “large *p* small *n*” problem has posed a significant challenge in the analysis and interpretation of genome-wide association studies (GWAS). The use of prior information to rank genomic regions and perform SNP selection could increase the power of GWAS. In this study, we propose the use of gene expression data from RNA-Seq of multiple tissues as prior information to assign weights to SNP, select SNP based on a weight threshold, and utilize weighted hypothesis testing to conduct a GWAS. RNA-Seq libraries from hypothalamus, duodenum, ileum, and jejunum tissue of 30 pigs with divergent feed efficiency phenotypes were sequenced, and a three-way gene x individual x tissue clustering analysis was performed, using constrained tensor decomposition, to obtain a total of 10 gene expression modules. Loading values from each gene module were used to assign weights to 49,691 commercial SNP markers, and SNP were selected using a weight threshold, resulting in 10 SNP sets ranging in size from 101 to 955 markers. Weighted GWAS for feed intake in 4,200 pigs was performed separately for each of the 10 SNP sets. A total of 36 unique significant SNP associations were identified across the ten gene modules (SNP sets). For comparison, a standard unweighted GWAS using all 49,691 SNP was performed, and only 2 SNP were significant. None of the SNP from the unweighted analysis resided in known QTL related to swine feed efficiency (feed intake, average daily gain, and feed conversion ratio) compared to 29 (80.6%) in the weighted analyses, with 9 SNP residing in feed intake QTL. These results suggest that the heritability of feed intake is driven by many SNP that individually do not attain genome-wide significance in GWAS. Hence, the proposed procedure for prioritizing SNP based on gene expression data across multiple tissues provides a promising approach for improving the power of GWAS.

## Introduction

The “large *p* small *n*” problem has posed a significant challenge in the analysis and interpretation of genome-wide association studies (GWAS; Diao and Vidyashankar, 2013). The problem refers to the scenario in statistical inference where the dimension of independent variables, *p*, is larger than the sample size, *n*. Typically in GWAS, the number of observations, *n*, is in the hundreds or thousands and the number of markers, *p*, is in the hundreds of thousands. Statistical procedures such as shrinkage estimation and variable selection are often employed to ensure the existence solutions in *large-p-small-n* regressions in GWAS (Fernando et al., 2017).

The most commonly used approach to GWAS is single-SNP analysis, where linear or logistic regression is performed separately for each SNP followed by multiple-testing correction. This standard single-step adjustment disregards prior knowledge of potentially noteworthy regions, and, as a result, tests of significance for SNP in such regions may be overly down-weighted due to the other relatively inconsequential SNP. Hence, using prior information to rank genomic regions and perform SNP selection could increase the power of GWAS.

Recent advances in statistical methodology have made it possible to incorporate prior information through weighted hypothesis testing (Genovese et al., 2006). Roeder et al. (2006) introduced a method which uses linkage analysis information to up- or down-weight SNP according to their prior likelihood of association with a trait of interest, and the resulting weighted P-values are used in the false discovery rate (FDR) procedure. A similar approach using expression quantitative trait loci (eQTL) information to weight SNP was proposed by Li et al. (2013).

Transcriptome sequencing (RNA-Seq) is a widely used technology for genome-wide transcript quantification, used to analyze gene expression patterns, and provide insight into the mechanisms underlying complex traits in livestock species. Genome-wide gene expression data from thousands of studies have been accumulating and made available through public repositories such as the Gene Expression Omnibus (GEO; Edgar et al., 2002). Recently, GWAS results have been interpreted by interrogating significant SNP for associations with gene expression data in livestock (Ballester et al., 2017; Fang et al., 2017; Kommadath et al., 2017; Cai et al., 2018; Deng et al., 2019). These studies have integrated GWAS and gene expression data post-GWAS. In this study, we propose the use of gene expression data from RNA-Seq of multiple tissues (hypothalamus, duodenum, ileum, and jejunum) as prior information to assign weights to SNP, select SNP based on a weight threshold, and utilize weighted hypothesis testing to conduct a GWAS for swine feed efficiency.

## Material and Methods

The U.S. Meat Animal Research Center (USMARC) Animal Care and Use committee reviewed and approved the use of animals in this study.

### Population

Feed intake and body weight gain were measured on cohorts of growing pigs reared at USMARC. All pigs were sired by either Landrace or Yorkshire boars sourced from 5 different genetic suppliers and produced out of Landrace-Yorkshire cross sows. Two different genetic suppliers are represented in each group of pigs. Pigs entered the barn at approximately 95 days of age at the beginning of the feeding trial and had *ad libitum* access to a standard corn/soybean meal-based diet that met or exceeded NRC requirements (NRC, 2012). Pigs in each cohort (196 per cohort) were assigned to one of 14 same-sex pens (14 pigs per pen) containing a single Feed Intake Recording System (FIRE) feeder (Osborne Industries, Inc., Osborne KS). After a 1-week adjustment period, daily feed intakes for each pig were recorded *via* the FIRE feeders and pigs were weighed at the beginning (d0) and end (d 42) of the feeding trial. Twenty-two cohorts of pigs had individual feeding events recorded.

Different numbers of animals from the population were used in different stages of the study. Feed intake data was collected on a total of 4,200 animals across the 22 cohorts. Four of these 22 cohorts (n = 784 animals) were used to select 30 animals with extreme feed efficiency phenotypes for RNA-Seq. Lastly, GWAS was performed using data from the 2,813 animals that were both genotyped and phenotyped. Detailed descriptions of each stage of the study are provided in subsequent sections.

### Sampling for RNA-Seq

Feed efficiency phenotypes were determined for each pig in four cohorts (n = 784 animals) by dividing average daily body weight gain (ADG) by average daily feed intake (ADFI) to determine the gain to feed ratio (Gain : Feed). From each cohort of pigs, a selection criterion was applied to select animals for further study that included ADG within ± 0.30 SD of the mean and the greatest and least ADFI (n = 7 or 8 per cohort). The descriptive statistics are presented in **Table 1**.

**Table 1:** Descriptive data of efficient and inefficient pigs (n = 30).^1,2^

	ADFI^3^, kg/d	Initial Weight, kg	Ending Weight, kg	ADG^4^, kg/d	Gain : Feed
Efficient (Low Intake)	2.08 ± 0.11	47.7 ± 2.8	88.8 ± 2.8	0.991 ± 0.04	0.458 ± 0.025
Inefficient (High Intake)	2.80 ± 0.11	51.1 ± 3.0	93.6 ± 3.2	1.025 ± 0.05	0.367 ± 0.16

^1^Animals selected included those with ADG within ± 0.30 SD of the mean and the greatest (inefficient) and least (efficient) ADFI.

^2^Data means ± SEM.

^3^Average daily feed intake.

^4^Average daily gain.

### Tissue Collection, RNA Isolation, and Sequencing

Tissue collection and RNA extraction were performed using the same procedures in each contemporary group. Sample collection time frame was consistent across cohorts. Pigs identified as high and low feed efficiency were euthanized with barbiturates in accordance with the American Veterinary Medical Association guidelines (AVMA, 2013). The head was removed, and the hypothalamus was collected and stored at -80°C as previously described (Thorson et al., 2017). One 3-cm segment of mid-jejunum and one 3-cm segment of mid-ileum were collected from pigs as previously described (Oliver et al., 2002). In addition, a 3-cm segment of duodenum was collected approximately 5-cm caudal of the cranial duodenal flexure.

Total RNA was isolated from the tissue samples using the RNeasy Mini Plus kit and QiaShredder columns (Qiagen, Valenci, CA, USA). Briefly, 800 ul of RLT buffer with β-mercaptoethanol were added to 50–100 mg of tissue samples and homogenized for 40 sec using an Omni Prep 6-station homogenizer (Omni International, Kennesaw, GA, USA). The homogenate was centrifuged in a QiaShredder column on full speed for 3 min. Genomic DNA was removed from the total RNA with the Qiagen RNeasy Plus mini-kit, according to the manufacturer's protocol, and the total RNA was eluted in 50 ul of RNase free water. Total RNA was quantified with a NanoDrop One spectrophotometer (Thermo Scientific, Wilmington, DE). The average 260/280 ratio was 2.05, with a range of 1.94–2.09. An Agilent Bioanalyzer RNA 6000 nano kit (Santa Clara, CA, USA) was used to determine the RNA integrity number (RIN). Only samples with a RIN of 8.0 and higher were used for the RNA sequencing. The average RIN was 9.1, with a range of 8.1–9.9.

Samples were prepared for RNA sequencing with the Illumina TruSeq Stranded mRNA High Throughput Sample kit and protocol (Illumina Inc., San Diego, CA, USA). The libraries were quantified with qRT-PCR using the NEBNext Library Quant Kit (New England Biolabs, Inc., Beverly, MA, USA) on a CFX384 thermal cycler (Bio-Rad, Hercules, CA, USA), and the quality of the library was determined with an Agilent Bioanalyzer DNA 1000 kit (Santa Clara, CA, USA). The libraries were diluted with Tris-HCL 10 mM, pH 8.5 with 0.1% Tween 20 to 4nM samples (Teknova, Hollister, CA. USA). All libraries were paired-end sequenced with 150 cycle high output sequencing kits for the Illumina NextSeq instrument. Bases of the paired-end reads for all sequenced libraries were identified with the Illumina BaseCaller, and FASTQ files were produced for downstream analysis of the sequence data. Sequence data is available for download from the National Center for Biotechnology Information (NCBI) Sequence Read Archive (SRA) BioProjects PRJNA528599 (hypothalamus), PRJNA528884 (duodenum), PRJNA529214 (ileum), and PRJNA529662 (jejunum).

### Sequence Data Processing

Read alignment of the RNA-Seq reads was carried out as follows. First, quality of the raw paired-end sequence reads in individual FASTQ files was assessed using FastQC (Version 0.11.5; www.bioinformatics.babraham.ac.uk/projects/fastqc), and reads were trimmed to remove adapter sequences and low-quality bases using the Trimmomatic software (Version 0.35; Bolger et al., 2014). The remaining reads were mapped to the Sscrofa 11.1 genome assembly using Hisat2 (Version 2.1.1; Kim et al., 2015) with its default parameters. The StringTie software (Pertea et al., 2015) was then used to calculate raw read counts for each of the 29,651 annotated genes in the NCBI Sscrofa 11.1 reference annotation (Release 106).

Filtering of lowly expressed genes and normalization of read counts was performed using a protocol that considers the multi-tissue structure of the data. First, raw read counts were normalized using the median of ratios normalization scheme from the DESeq2 software package (Love et al., 2014), where read counts are divided by sample-specific size factors determined by median ratio of gene counts relative to the geometric mean per gene. A normalized gene expression matrix was constructed for each tissue, and the arithmetic mean of expression values across samples within each tissue was computed. Genes with mean normalized expression < 100 in all 6 tissues were removed from further analysis.

### Three-Way Clustering *Via* Constrained Tensor Decomposition to Detect Gene Expression Modules

Three-way clustering of multi-tissue, multi-individual gene expression data was performed using an adaption of the method described by Wang et al. (2017). Gene expression measurements for the four tissues were organized into a 3-way array, or order-3 tensor, with gene, individual, and tissue modes. That is, the input to the algorithm was an order-3 tensor given by, $\Omega =⟦\omega ijk⟧\in {\mathbb{R}}^{n_{G}\times n_{I}\times n_{T}}$, where ω_*ijk*_, denotes the normalized gene expression value for gene *i* in individual *j* in tissue *k*, *n*_*G*_ the number of genes, *n*_*I*_ the number of individuals, and *n*_*T*_ the number of tissues. The tensor Ω was then decomposed into a sum *R* of rank-1 components,

(1) $$\Omega =\sum_{r=1}^{R}\lambda_{r}G_{r}⊗I_{r}⊗T_{r}+\varepsilon ,$$

where λ_1_ ≥ λ_1_ ≥ … ≥ λ_*R*_ ≥0 are singular values in decreasing order, and ***G***_*r*_, ***I***_*r*_, and ***T***_*r*_ are norm-1 singular vectors that indicate the relative contribution of each gene, individual, and tissue to the *r*-th component, respectively, and ε = [*E*_*ik*_] is a noise tensor with each entry *E*_*ik*_ i.i.d. *N*(0,σ^2^).

Complete details of the algorithm used for tensor decomposition can be found in Wang et al. (2017). Briefly, the successive rank-1 approximation to Ω is determined by iteratively solving the following minimization problem:

(2) $$\underset{\lambda_{r},\;G_{r},I_{r},T_{r}}{\text{minimize}}\|\text{Ω}-\;\lambda_{r}G_{r}⊗I_{r}⊗T_{r}{\|}_{F},$$

$$\text{subject to }G_{r}{\|}_{2}=\;\|I_{r}{\|}_{2}=\;\|T_{r}{\|}_{2}=1,$$

where ‖·‖_*F*_ is defined entry-wise as ${\text{Ω}}_{F}=\;\sqrt{\sum_{i=1}^{n_{G}}\sum_{i=1}^{n_{I}}\sum_{i=1}^{n_{T}}\omega_{ijk}^{2}}$. At each iteration, we imposed one of two conditions, either ***T***_*r*_ ≥ 0 or ***T***_*r*_ ≤ 0, by thresholding values in to 0. The appropriate sign of was selected to maximize ***T***_*r*_. This constraint on eases the interpretation of the interaction at the tissue level. Non-zero tissue loading values indicate that the module is “active” in the tissue. Without constraining values in to a single sign, it is possible (in fact likely) that contains two expression modules, one for the tissues with positive loading values and one for the tissues with negative loading values. Consequently, gene and individual loadings become less informative since they cannot be explicitly assigned to either the positive or negative loading module. Note that the constraint on used in this work is slightly different that of Wang et al. (2017), where they imposed strict non-negativity on ***T***_*r*_.

Genes with large values in ***G*** *_r_* exhibit strong relationships with individuals and tissues in the *r*-th component, while these relationships are stronger in the individuals with larger ***I*** *_r_*-values and tissues with larger (in absolute value) ***T*** *_r_*-values. The loading vectors ***G*** *_r_*, ***I*** *_r_*, and ***T*** *_r_* will be referred to as eigen-genes, eigen-individuals, and eigen-tissues, respectively, throughout the remainder of the manuscript.

### Gene Ontology Enrichment Analysis

Enrichment analysis of gene ontology GO terms was performed using the PANTHER classification system (Version 14.1; Mi et al., 2016). PANTHER's implementation of the binomial test of overrepresentation with the default Ensembl *Sus scrofa* GO annotation as background was utilized. Data from PANTHER was considered statistically significant at FDR-corrected *P* ≤ 0.05.

### Characterization of Gene Expression Modules

GO enrichment analysis was performed on the top genes within each expression module, where the top genes in module *r* were defined as genes having a loading value in ***G***_*r*_ greater than a specified cutoff value, *c*, which controls the significance level. A permutation-based approach was used to determine *c* with an arbitrarily selected significance level of *α* = 0.005. One hundred null tensors were generated by randomly and independently permuting gene expression values for every individual-tissue pair. That is,

$$\Omega^{\text{null}}\left(G,\text{ individual }j,\text{ tissue }k\right)≝\Omega \left(\mathrm{PG},\text{ individual }j,\text{ tissue }k\right),$$

where G denotes the original set of gene expression values and PG denotes the permutated gene expression values. Each null tensor was decomposed, and their eigen-genes were used to represent the null distribution of gene expression values within each module. The cutoff value for module *r*, *c*_*r*_, was the 99.5-percentile of the empirical distribution of $G_{r}^{\text{null}}$.

### Proportion of Variance in Individual Loadings

Sources of variation in individual loadings were analyzed by fitting the following linear model:

(3) $$I_{j}=\beta_{1}+\beta_{2}{\text{ADFI}}_{j}+\beta_{3}{\text{CG}}_{j}+\beta_{4}{\text{Gender}}_{j}+\varepsilon_{j},$$

where ADFI denotes average daily feed intake, CG denotes contemporary group, $I={\left(I_{1},\ldots ,I_{n_{I}}\right)}^{T}$, and *ε*_*j*_~*N*(0, *σ* ^2^) for all *j* = 1, 2,…, *n*_*I*_. After the model was fit, the proportion of variance explained by each covariate (ADFI, contemporary group, and gender) was calculated using ANOVA.

### Tensor Projection for Identifying ADFI-Associated Genes

Using the notation from above, let $\text{Ω}\in \;{\mathbb{R}}^{n_{G}\times n_{I}\times n_{T}}$ denote the expression tensor and $\left\{T_{r}\in {\mathbb{R}}^{n_{T}}\right\}$ be the set of eigen-tissues from the tensor decomposition. Let Ω(·, ·, ***T***_***r***_) be the tensor projection of Ω through the eigen-tissue $T_{r}={\left(T_{r,1},...,T_{r,n_{T}}\right)}^{T}$, i.e.,

(4) $$\text{Ω}\left(\cdot ,\cdot ,T_{r}\right)=\sum_{k=1}^{n_{T}}T_{r,k}\;\Omega \left(\cdot ,\cdot ,k\right).$$

Then, the following linear model was used for each gene tested,

(5) $$\text{Ω}\left(\text{test gene},\;\cdot ,T_{r}\right)=\;\beta_{1}1+\;\beta_{2}ADFI+\;\beta_{3}CG+\beta_{4}Gender+\varepsilon ,\;\;$$

where *ε*_*j*_~* N*(**0**, *σ* ^2^ ***I***). The ADFI-effect was assessed by testing $H_{0}:\beta_{2}=0$ against $H_{\alpha }:\beta_{2}\neq 0$.

### Phenotypic Data Collection for Genetic Association Analysis

Twenty-two cohorts of 196 pigs had individual feeding events recorded in a building fitted with Osbourne FIRE Feeders. The animals and facilities were previously described in Section 2.1. Records were removed for animals with incomplete data due to one of the following reasons: animal removed from the study due to health, failure of the electronic ID eartag, or failure of the FIRE Feeder for a majority of the test. As a result, 4,200 animals remained in the study. Aberrant feeding events were removed if they did not conform to a logical length of meal time (1 sec < meal time < 3,600 sec), amount of feed consumed (20 g < feed consumed < 3 kg), and consumption rate (rate < 2 kg/min). Once aberrant feeding events were removed, feeding parameters were computed for each pen and day of test to determine if a feeder was not operating properly. Statistics used to remove a pen x day included number of aberrant feeding events recorded, amount of feed distributed, and total number of events for each day. After all suspicious records were removed, the amount of feed consumed by each pig for each day of test was calculated, resulting in a total of 164,660 records of the 184,800 possible daily intake records.

Data were analyzed with WOMBAT (Version 17-07-2017; Meyer, 2007) fitting a random regression mixed model. Fixed effects fitted were gender (barrow or gilt) and a combined group-pen effect. Day on test was fit as the independent variable using a cubic Legendre polynomial, and animal was fitted as a random effect. A cubic Legendre polynomial was selected as it dramatically improved the log likelihood of the model over a quadratic Legendre regression and only marginal improvements were seen when evaluating higher order polynomials. Random regression coefficients were projected to individual daily intake for each of the days on test, to fill the missing intake records and adjust for fixed effects. Daily projections were summed to obtain adjusted test intake for each individual.

### Genotypic Data Collection for Genetic Association Analysis

Tail samples were collected on all pigs and stored at −20°C. Genomic DNA was extracted using the WIZARD genomic DNA purification kit according to the manufacturer's protocol (Promega Corp., Madison, WI, USA). Genotyping was conducted using three platforms: the NeoGen Porcine GGPHD chip (GeneSeek, Lincoln, USA), Illumina Porcine SNP60 v2 chip (Illumina, Inc., San Diego, USA), and NeoGen GGP-Porcine chip (GeneSeek, Lincoln, USA).

### Genetic Association Analysis

Ancestors of the pigs having intake records were identified from USMARC pedigree records to create a 7,009 animal pedigree. Phenotyped pigs and their ancestors genotyped with a SNP assay, Illumina Porcine SNP60 v1 or v2 (Illumina, Inc., San Diego, USA), Illlumina Porcine SNP50 (Illumina, Inc., San Diego, USA), NeoGen GGP-Porcine chip (GeneSeek, Lincoln, USA), and NeoGen Porcine GGPHD chip (GeneSeek, Lincoln, USA) were identified. The SNP were ordered according to the Sscrofa11.1 genome assembly and available pedigree was used to impute genotypes to 49,695 SNP from at least one assay for the 4,632 genotyped animals (2,813 phenotyped, 1,819 ancestors) using findhap (VanRaden et al., 2013).

Following VanRaden (2008), weighted genomic relationship matrices (G), were constructed as

(6) $$G=\frac{M^{*'}M^{*}}{2{\text{Σ}}_{i=1}^{m}p_{i}\left(1-p_{i}\right)},$$

where m is the number of SNP, p_i_ the frequency of the B allele for the i^th^ SNP, and M^*^ a centered genotype matrix (M) weighted by a diagonal matrix of weighting factors (D)

(7) $$M^{*}=MD.$$

Genomic relationship matrices were constructed for equally weighted SNP (D = m x m identity matrix) as well as for gene-centric weightings. Weights for SNP within gene boundaries were calculated as −*log*_10_(*P*), where P denotes the P-value obtained from testing the ADFI-effect in Equation (5) in the gene module of interest. If a SNP did not reside in a gene, it was assigned a weight of zero.

For a given weight threshold, t, three G for each of the 10 sets of gene weightings were evaluated: (1) a weighted analysis with all SNP where all SNP had non-zero weightings (min = 0.00001), (2) an unweighted analysis using only SNP with weight > *t*, and (3) a weighted analysis using only SNP with weight > *t*. Arbitrary thresholds of *t* = 2 and *t* = 5 were evaluated.

The average information restricted maximum likelihood (AIREML) algorithm implemented in WOMBAT was used to estimate heritability (h^2^) of test intake attributable to pedigree relationships and each weighted genomic relationship matrix. Phenotypic variance should remain constant; all estimates of phenotypic variance from these data using different unweighted G were similar. Weighted G resulted in additive variance estimates much greater than phenotypic variance from unweighted G, and residual variances were similar to estimates using unweighted G. Assuming the residual variance estimate is appropriate for variation not explained by weighted G and phenotypic variance equal to that estimated with unweighted G, the amount of variation explained by weighted G should be the difference between phenotypic variance from unweighted G and residual variance from weighted G, and corrected heritability that difference divided by phenotypic variance. That is,

$$h_{w}^{2}=\;\frac{Var\left(P_{u}\right)-Var\left(E_{w}\right)}{Var\left(P_{u}\right)}$$

where *P*_*u*_ denotes the phenotypic variance from unweighted G, and *E*_*w*_ denotes the residual variance from weighted G.

After convergence, effects of individual SNP were estimated for each genomic relationship matrix. Following Wang et al. (2012),

(8) $$\hat{a}=M^{*'}{\left[M^{*}M^{*'}\right]}^{-1}\hat{u_{g}},$$

where $\hat{a}$ is a vector of SNP effect estimates and $\hat{u_{g}}$ the vector of animal effects predicted for each genotyped animal. Z-scores were computed standardizing $\hat{a}$ to a mean of zero and variance of one:

$$Z_{i}=\;\frac{a_{i}-\;\bar{a}}{SD\left(\hat{a}\right)}$$

where $\bar{a}$ and $SD\left(\hat{a}\right)$ denote the mean and standard deviation of $\hat{a}$, respectively.

## Results

### Sequencing, Read Mapping, and Gene Expression

RNA-Seq libraries from hypothalamus, duodenum, ileum, and jejunum tissue of 30 pigs with divergent feed efficiency phenotypes were sequenced, generating over 7.4 billion 75-bp paired-end reads, with an average of 61.8 million reads per library (**Table 2**). After adapter removal and read trimming, the resulting high-quality reads were mapped to the Sscrofa 11.1 genome assembly (NCBI accession AEMK00000000.2) with an average 98.6% read mapping rate per library. Sequencing statistics for individual libraries are given in **Table S1**.

**Table 2:** Summary of sequencing statistics by tissue.

Tissue	Total number reads	Mean number reads per library	Mean read mapping % per library
Hypothalamus	2,014,157,388	67,138,579.6	96.91%
Duodenum	1,653,423,084	55,114,102.8	98.25%
Jejunum	1,809,768,258	60,325,608.6	99.59%
Ileum	1,942,804,030	64,760,134.3	99.47%
			
All	7,420,152,760	61,834,606.3	98.55%

Normalized gene expression values were computed for the 29,651 annotated genes in the porcine genome, and lowly expressed genes across the six tissues were removed, resulting in a set of 19,365 genes to be used in downstream analyses. **Table 3** shows the number of genes expressed in each of the tissues, where a gene is considered expressed if normalized expression ≥ 100 in at least fifteen (half) of the libraries in the tissue. An average of 13,351 genes were expressed per tissue.

**Table 3:** Summary of expressed genes by tissue.

Tissue	Total number of genes expressed^1^
Hypothalamus	14,205
Duodenum	12,824
Jejunum	12,734
Ileum	13,640

^1^Genes defined as expressed if normalized expression ≥ 100 in at least 15 libraries.

### Expression Modules Across Individuals and Tissues

A three-way gene x individual x tissue clustering analysis was performed, using constrained tensor decomposition, to obtain a total of 10 gene expression modules.

#### Module I – Shared, Global Expression

**Figure 1:**
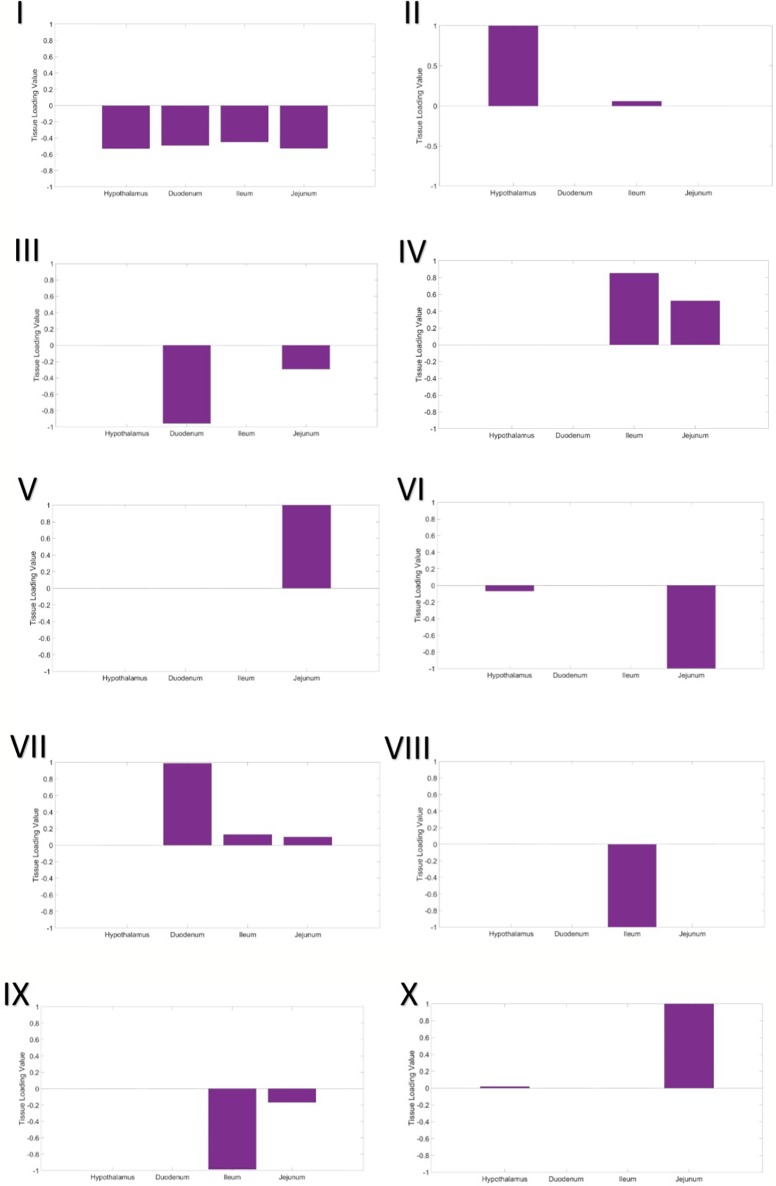
Tissue loading values for Modules I–X from the tensor decomposition.

In the first gene expression module, the eigen-tissue and eigen-individual loading distributions are essentially flat (**Figure 1I**). Hence this module captures baseline, global gene expression common to all samples in all tissues. Enrichment analysis showed that many GO terms related to basic eukaryotic cell activities were enriched in the set of 1,307 top genes, including ion binding, protein binding, nucleotide binding, and transport (**Table S2**).

#### Module II – Hypothalamus

The second gene expression module clearly separated the hypothalamus from the intestinal tissues (**Figure 1II**). In the eigen-individual, more of the proportional variance in loading values was explained by ADFI than contemporary group or gender (6.8% compared to 1.15% and 0.96%, respectively; **Table 4**). The top 130 genes were enriched for functions related to nucleotide binding, protein binding, ion binding, hydrolase activity, and glutamate transporter activity.

**Table 4:** Proportional variance in individual loading values explained by average daily feed intake (ADFI), contemporary group, and gender in each of the modules obtained from the tensor decomposition.

Module	ADFI	Cont. Group	Gender
I	2.02%	3.46%	3.28%
II	6.80%	1.15%	0.96%
III	5.58%	0.00%	8.13%
IV	6.05%	10.53%	0.01%
V	0.00%	10.98%	0.67%
VI	0.36%	6.08%	0.14%
VII	0.00%	18.01%	0.42%
VIII	1.97%	1.47%	2.11%
IX	2.98%	10.09%	0.00%
X	5.05%	21.22%	0.26%

#### Module III – Proximal Small Intestine

The third component captures expression specific to tissues in the proximal small intestine, the duodenum and jejunum. The eigen-tissue is primarily driven by the duodenum (**Figure 1III**). A moderate amount of variation among individuals was explained by both gender (8.13%) and ADFI (5.58%), while the variance explained by contemporary group was negligible (~ 0%). A total of 88 genes passed the thresholding to be considered a top gene in the module. These genes were primarily enriched for binding GO terms, including G protein-coupled receptor binding, sulfur compound binding, carbohydrate derivative binding, bile acid binding, cytoskeletal protein binding, ubiquitin protein ligase binding, nucleotide binding, and metal ion binding. Nearly 83% (73/88) of the top genes were also identified as top genes in the hypothalamus expression module (Module II).

#### Module IV – Distal Small Intestine (positive loadings)

The fourth gene expression module was comprised of the distal small intestinal tissues, the jejunum and ileum, with the ileum being the main driver (larger loading value; **Figure 1IV**). Although contemporary group explained the largest amount of proportional variance (10.53%), a moderate amount of variation, 6.05%, was explained by ADFI. Top genes in the module were enriched for functions related to peptide transport, lipid transport, chemokine receptor binding, hydrolase activity, bile acid binding, peptidase inhibition, and ion binding. Only one of the top genes, *COX1*, overlapped with the top genes from Module II, while 15 genes from Module III's top set were overlapped.

#### Module V – Jejunum

Expression in the jejunum tissue was captured in the fifth component (**Figure 1V**). Contemporary group was the only covariate to account for more than 1% of the variation among individuals. GO analysis of the 121 top genes identified that translation regulation, RNA binding, fatty acid binding, and rRNA binding were significantly enriched.

#### Module VI – Jejunum and Hypothalamus (negative loadings)

The sixth module included the hypothalamus and the jejunum in the eigen-gene, with the jejunum tissue having a much stronger effect (**Figure 1VI**). Again, contemporary group was the main covariate explaining individual loading value variation, as it explained approximately 6% of the variation and ADFI and gender each explained less than 1%. No GO terms were significantly enriched in the set of top genes.

#### Module VII – Small Intestine

Expression in all three parts of the small intestine, the duodenum, jejunum, and ileum, was captured in the seventh module. The duodenum was the most significant driver, while the jejunum and ileum had very similar loading values (**Figure 1VII**). Once again, variation in loading values in the eigen-individual was predominantly explained by contemporary group. The GO term CMP-N-acetylneuraminate monooxygenase activity was significantly enriched in the top genes.

#### Module VIII – ileum

Ileum gene expression was highlighted in the eighth component (**Figure 1VIII**). Variation between individual loading values was not well-explained by any of the covariates in the model, ADFI (1.97%), contemporary group (1.47%), and gender (2.11%). No GO terms were significantly enriched in the set of top genes. Additionally, no top genes were overlapped with those from Module IV, which was also driven by gene expression in the ileum.

#### Module IX – distal small intestine (negative loadings)

The fourth gene expression module was comprised of the distal small intestinal tissues, the jejunum and ileum (**Figure 1IX**). It should be noted that this module corresponds to negative loading values for the tissues, while the results in Module IV corresponded to positive loading values. Similar to Module IV, ileum was the main driver of expression in the module, and contemporary group explained the largest amount of variation between individual loadings. However, none of the top genes were found to be top genes in Module IV, and no GO terms were significantly enriched.

#### Module X – jejunum and hypothalamus (positive loadings)

The sixth module included the hypothalamus and the jejunum in the eigen-gene, with the jejunum tissue having a much stronger effect (**Figure 1X**). This module corresponds to positive loading values in the eigen-tissue, while Module VI gave the results for negative loading values. A larger amount of variation among individuals was explained by covariates in the model than that from Module IV, contemporary group explained 21.22% and ADFI explained 5.05%. The GO term CMP-N-acetylneuraminate monooxygenase activity was significantly enriched in the top genes. There was no overlap between the set of top genes and the top genes from Module IV.

### Genetic Association Analysis

For each of the gene modules, three genetic association analyses were conducted: (1) a weighted analysis with all SNP, (2) an unweighted analysis using only SNP with weight > 5, and (3) a weighted analysis using only SNP with weight > 5. Removal of low weight SNP resulted in SNP sets ranging in size from 101 to 944 markers (**Table 5**). Results from these analyses are shown in **Tables 5** and **6**. Utilization of all 49,691 SNP with pedigree and genomic relationships resulted in heritabilities of 0.366 and 0.269, respectively. In general, applying SNP weights derived from each of the gene models resulted in heritabilities that remained close to those derived from the unweighted pedigree and genomic models (**Table 7**).

**Table 5:** Heritability estimates for feed efficiency from unweighted genome-wide association studies (GWAS) utilizing SNP with weight > 5.

Data Set	Heritability (h^2^)	Standard Error (SE)	# SNP	h^2^/# SNP
Module I	0.069	0.016	183	3.77E-04
Module II	0.062	0.015	204	3.03E-04
Module III	0.061	0.016	145	4.21E-04
Module IV	0.088	0.018	944	9.32E-05
Module V	0.088	0.018	536	1.64E-04
Module VI	0.040	0.012	192	2.08E-04
Module VII	0.045	0.013	101	4.46E-04
Module VIII	0.081	0.017	528	1.53E-04
Module IX	0.081	0.017	624	1.30E-04
Module X	0.015	0.014	296	5.07E-05

**Table 6:** Heritability estimates for feed efficiency from weighted genome-wide association studies (GWAS) utilizing SNP with weight > 5.

Data Set^1^	Heritability (h^2^)^2^	Standard Error (SE)	# SNP	h^2^/# SNP
Module I	0.106	0.049	183	5.81E-04
Module II	0.099	0.050	204	4.86E-04
Module III	0.094	0.053	145	6.52E-04
Module IV	0.137	0.0374	944	1.45E-04
Module V	0.156	0.026	536	2.91E-04
Module VI	0.073	0.072	192	3.82E-04
Module VII	0.081	0.066	101	7.98E-04
Module VIII	0.131	0.036	528	2.49E-04
Module IX	0.128	0.040	624	2.04E-04
Module X	0.089	0.061	296	3.00E-04

^1^SNP weights derived from indicated gene module.

^2^Heritability estimates were corrected using the difference between the phenotypic variance estimated with the unweighted G and residual variance estimated with each weighted G.

**Table 7:** Heritability estimates for feed efficiency from weighted genome-wide association studies (GWAS) utilizing all SNP.

Data Set^1^	Heritability (h^2^)^2^	Standard Error (SE)	# SNP	h^2^/# SNP
Pedigree (Unweighted)	0.366	0.045	49,691	7.37E-06
Genomic (Unweighted)	0.269	0.031	49,691	5.42E-06
Module I	0.270	0.035	49,691	5.44E-06
Module II	0.271	0.037	49,691	5.46E-06
Module III	0.269	0.028	49,691	5.41E-06
Module IV	0.273	0.040	49,691	5.49E-06
Module V	0.272	0.038	49,691	5.46E-06
Module VI	0.270	0.034	49,691	5.43E-06
Module VII	0.269	0.026	49,691	5.40E-06
Module VIII	0.272	0.039	49,691	5.49E-06
Module IX	0.273	0.039	49,691	5.49E-06
Module X	0.270	0.035	49,691	5.44E-06

^1^SNP weights derived from indicated gene module.

^2^Heritability estimates were corrected using the difference between the phenotypic variance estimated with the unweighted G and residual variance estimated with each weighted G.

The removal of SNP with weight < 5 and leaving SNP unweighted in the model decreased performance in all 10 modules (**Table 5**), i.e., heritabilities were below those of the pedigree and unweighted models. Removal of SNP with weight < 5 and utilizing the SNP weights in the model increased performance from the unweighted case in all ten modules, but overall heritability was still lower than that obtained from using all SNP (**Table 6**).

Output from the association analyses for feed intake is shown in **Table S3**. A total of 36 unique significant SNP associations were identified across the ten gene modules, while 2 only SNP were significant in the standard analysis using all 49,691 SNP with no SNP weights. Neither of the 2 SNP identified in the unweighted analysis were identified in the weighted analyses. The number of significant SNP identified in each module's analysis ranged from 0 to 22, with Modules I, II, VI, VII, and X having only no significant SNP and Module III having 22 significant SNP. For the weighted analyses, significant SNP were identified on chromosomes SSC 2, 4, 5, 7, 8, 9, 13, 14, 15, 18, and X, with SSC 9 and SSC 8 having the largest numbers of significant SNP, 12 and 6, respectively.

## Discussion

The most widely used approach to GWAS has been to assign equal prior probability of association to all sequence variants tested. Recent findings suggest that incorporating prior information can increase the power for identifying associations. Such prior information can be obtained from several different sources, including but not limited to linkage analysis (Roeder et al., 2006), gene expression (Li et al., 2013; Gamazon et al., 2015; Gusev et al., 2016; Xu et al., 2017), and functional annotation of variants (Sveinbjornsson et al., 2016). In this work, we present a methodology that exploits multi-tissue transcriptional data from a small set of individuals with extreme phenotypes to assign SNP weights for a GWAS on an expanded set of phenotyped individuals. It has been shown that any set of nonnegative weights can guarantee substantial power gain if the weights are informative and little power loss if the weights are uninformative (Genovese et al., 2006). Hence, the weighting procedure is robust to the informativeness of the weights.

We applied our method to identify genetic markers associated with feed intake in swine. The gut-brain axis is comprised of bidirectional communication between the central and enteric nervous systems, linking cognitive centers of the brain with peripheral intestinal functions. The gut-brain axis modulates short-term satiety and hunger responses to regulate the delivery of nutrients and transit of nutrients through the gastrointestinal tract (Hussain and Bloom, 2012). RNA-Seq was performed on tissues involved in the gut-brain axis, including hypothalamus, duodenum, ileum, and jejunum, originating from pigs with extreme feed intake phenotypes. A tensor decomposition method, which performs three-way clustering across genes, tissues, and individuals, was used to identify gene expression modules that were either common to all tissues and individuals or exclusive to particular tissue/individual combinations.

The top ten gene modules from the tensor decomposition were considered. Note that since the clustering algorithm generates expression modules *via* successive rank-1 approximations, if more expression modules were desired the algorithm could simply be applied to the residual tensor. Module I captured baseline, global gene expression common to all samples in all tissues, indicated by the flat distributions of the eigen-tissue and eigen-individual loading values. Other gene modules captured expression specific portions of the gut-brain axis, including the hypothalamus, the proximal and distal small intestine, the entire small intestine, and the individual components of the small intestine.

A tensor projection model was used to identify ADFI-associated genes within each of the ten modules. The P-values obtained from testing the ADFI-effect were used to weight SNP in order to conduct a weighted GWAS. P-values were chosen over regression coefficients for weighting in order to rank SNP according to the significance of their respective genomic regions rather than simply an effect size. Results from both the weighted and unweighted analyses are shown in **Tables 7**–**9**. Preliminary analyses using weighted SNP revealed what appeared to be inflated estimates of heritability. There was substantially less change in residual variance estimates, indicating that inflated heritability was not a result of explaining substantially more phenotypic variation with the weighted G, but an artifact of weighted G resulting in inflated additive and phenotypic variance estimates. Phenotypic variance should remain constant, so heritability estimates were corrected using the difference between the phenotypic variance estimated with the unweighted G and residual variance estimated with each weighted G.

**Table 8:** Heritability estimates for feed efficiency from unweighted genome-wide association studies (GWAS) utilizing SNP with weight > 2.

Data Set	Heritability (h^2^)	Standard Error (SE)	# SNP	h^2^/# SNP
Module I	0.209	0.028	5,915	3.53E-05
Module II	0.182	0.026	5,259	3.35E-05
Module III	0.139	0.022	1,992	6.98E-05
Module IV	0.221	0.027	7,290	3.03E-05
Module V	0.184	0.025	4,442	4.14E-05
Module VI	0.175	0.025	4,311	4.06E-05
Module VII	0.148	0.022	1,951	7.59E-05
Module VIII	0.229	0.028	9,283	2.47E-05
Module IX	0.225	0.028	7,932	2.84E-05
Module X	0.183	0.025	4,250	4.31E-05

**Table 9:** Heritability estimates for feed efficiency from weighted genome-wide association studies (GWAS) utilizing SNP with weight > 2.

Data Set^1^	Heritability (h^2^)^2^	Standard Error (SE)	# SNP	h^2^/# SNP
Module I	0.217	0.036	5,915	3.66E-05
Module II	0.205	0.035	5,259	3.90E-05
Module III	0.166	0.043	1,992	8.31E-05
Module IV	0.255	0.026	7,290	3.50E-05
Module V	0.222	0.028	4,442	5.00E-05
Module VI	0.200	0.037	4,311	4.65E-05
Module VII	0.174	0.041	1,951	8.93E-05
Module VIII	0.250	0.030	9,283	2.69E-05
Module IX	0.238	0.031	7,932	3.00E-05
Module X	0.208	0.034	4,250	4.90E-05

^1^SNP weights derived from indicated gene module.

^2^Heritability estimates were corrected using the difference between the phenotypic variance estimated with the unweighted G and residual variance estimated with each weighted G.

There was a common pattern to the change in heritability estimates as the SNP prioritization changed. When using all 50K unweighted SNP, the heritability increased from 0.269 using genomic relationships to 0.366 using pedigree. In all ten modules, the use of weighted SNP restricted to those with weight > 2 resulted in a heritability slightly lower, but comparable to that from the usual unweighted, genomic model. Randomization of SNP weights (**Table S4**) resulted in nearly the same overall and average per SNP heritabilities, suggesting that the weighting threshold may be suboptimal.

To investigate if a more stringent SNP weight threshold could increase model performance, SNP with weight < 5 were removed from the analysis. This resulted in an average 21-fold drop in the number of SNP included in each analysis (**Table 5**). Although overall heritability estimates were lower than those obtained using SNP with weight > 2, the heritability per SNP increased. Additionally, in most modules, both overall and per SNP heritabilities were higher than those obtained when the SNP weights were randomized. The numbers of SNP (101 < p < 944) in these analyses were smaller than the number of animals (n = 4,200), eliminating the ‘p greater than n' problem. Hence, applying a more stringent threshold results in a more informative set of SNP. Note the weight threshold values of 2 and 5 were chosen arbitrarily. Additional investigation will be needed to determine the optimal weight threshold for SNP inclusion, but this was outside the scope of this study.

Across the ten gene modules (weight > 5), 36 unique SNP were identified as having significant effects, while only 2 SNP were significant in the unweighted analysis utilizing all 50K SNP. Neither SNP from the unweighted analysis resided in known QTL related to swine feed efficiency (feed intake, average daily gain, and feed conversion ratio) compared to 29 (80.6%) in the weighted analyses, with 9 SNP being located in feed intake QTL (**Table S4**). Additionally, many of the genes harboring significant SNP have been identified in previous studies as candidate genes related to feed efficiency in several species (**Table S5**). In particular, the genes *ROBO2* (2 SNP), PLA2G4A (4 SNP), and *MEGF10* (1 SNP) were previously identified as candidate genes for residual feed intake and feed conversion ratio in swine (Ding et al., 2018; Horodyska et al., 2019). Hence, the results from this study suggest that a considerable proportion of heritability of feed intake is driven by many SNP that individually do not attain genome-wide significance in GWAS and therefore support a highly polygenic architecture for feed intake.

Our integrated methodology, at present, is obviously partial to genotyped SNP within genes. Because most available biological resources are biased toward genes, SNP pertaining to known genes likely have more relevant prior information. Consequently, the resulting weights may be more effective for associated SNP residing in or close to known genes. Therefore, results derived from our method can still be informative regardless of their intrinsic bias. Future work will focus on extending the scope of the tensor decomposition step to leverage data from other genomic sources, including but not limited to expression of non-coding RNA, miRNA expression, transcription factors, methylation targets, and miRNA binding. Additionally the method will be extended to prioritize variants from whole genome sequencing for assay development based on functional effects.

## Data Availability Statement

The datasets generated for this study can be found in the NCBI SRA Accession Numbers PRJNA528599, PRJNA528884, PRJNA529214, and PRJNA529662.

## Author Contributions

BK conceived of the study, and all authors participated in its design and coordination. WO and AP were involved in the acquisition of data, and BK, WS, LK, and GR performed data analyses. BK drafted the manuscript, and AP, WO, LK, WS, and GR contributed to the writing and editing. All authors read and approved the final manuscript.

## Disclaimer

Mention of trade names or commercial products in this publication is solely for the purpose of providing specific information and does not imply recommendation or endorsement by the U.S. Department of Agriculture. The U.S. Department of Agriculture (USDA) prohibits discrimination in all its programs and activities on the basis of race, color, national origin, age, disability, and where applicable, sex, marital status, familial status, parental status, religion, sexual orientation, genetic information, political beliefs, reprisal, or because all or part of an individual’s income is derived from any public assistance program. (Not all prohibited bases apply to all programs.) Persons with disabilities who require alternative means for communication of program information (Braille, large print, audiotape, etc.) should contact USDA’s TARGET Center at (202) 720-2600 (voice and TDD). To file a complaint of discrimination, write to USDA, Director, Office of Civil Rights, 1400 Independence Avenue, S.W., Washington, D.C. 20250-9410, or call (800) 795-3272 (voice) or (202) 720-6382 (TDD). USDA is an equal opportunity provider and employer.

## Conflict of Interest

The authors declare that the research was conducted in the absence of any commercial or financial relationships that could be construed as a potential conflict of interest.

## Supplementary Material

Table S1 Sequencing statistics.

Table S2 Results from Gene Ontology (GO) analysis in each gene module.

Table S3 Significant SNP in association analysis and QTL and genes overlapped.

Table S4 Results of GWAS for SNP weight randomization.

Table S5 Associations identified in previous literature for significant SNP.
